# Antimicrobial stewardship in Australian health care: 2018

**DOI:** 10.1093/jacamr/dlz010

**Published:** 2019-04-12

## Abstract

**Graphical Abstract:**
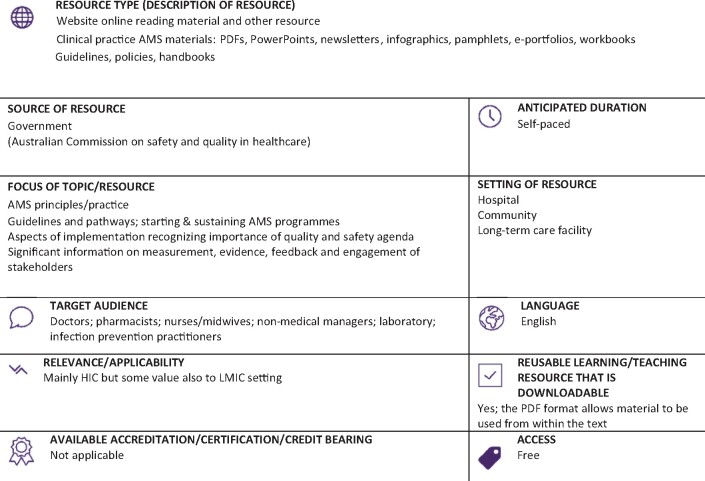

**Resource web link:** **https://www.safetyandquality.gov.au/wp-content/uploads/2018/05/AMSAH-Book-WEB-COMPLETE.pdf** (Full classification scheme available at: http://bsac.org.uk/wp-content/uploads/2019/03/Educational-resource-review-classification-scheme.pdf)

**WHO region and country (World Bank):** Australasia, Australia (HIC)

## Peer review commentary

This is a comprehensive excellent antimicrobial stewardship (AMS) resource, a book available in downloadable PDF format with lots of good illustrative figures, tables, case histories and references to activities/resources for Australian clinicians but also of some relevance to those outside Australia. Produced in collaboration with a range of reputable stakeholders in Australia it represents an invaluable resource that encompasses evidence and extensive experience of developing, delivering and measuring stewardship practice across the Australian healthcare systems. Relatively few available AMS resources encompass the One Health system approach and this is a commendable aspect of this resource.

The book contains just about most things you would need to deliver AMS but I was particularly struck by coverage given to roles of nurses and infection prevention practitioners, how business cases can be built (often a challenge for busy clinicians), how to engage consumers and tackle conflicts of interest and relationships with pharma. The material covered tries to align community practice with hospital practice giving a more seamless approach to AMS. All of this is supported with pragmatic case studies, audits and so on. The section on IT and electronic decision support is particularly a strong feature of Australian AMS and the importance of using technologies such as telehealth is of particular relevance where there are remote and rural populations.

Immersion of AMS within quality and safety of standards for Australian healthcare is well highlighted and something others can learn from.

The resource would benefit more from explaining how implementation science can support practice and sustainability and perhaps signposting to other educational resources that could be accessed freely by the global community would be helpful. Although the resource is aimed at the Australian healthcare system it has global value for other HICs and LMICs but I am not sure of value to LICs.

I would highly commend this resource to all particularly as the 2018 version is a considerable update on the previous one of 2014.

